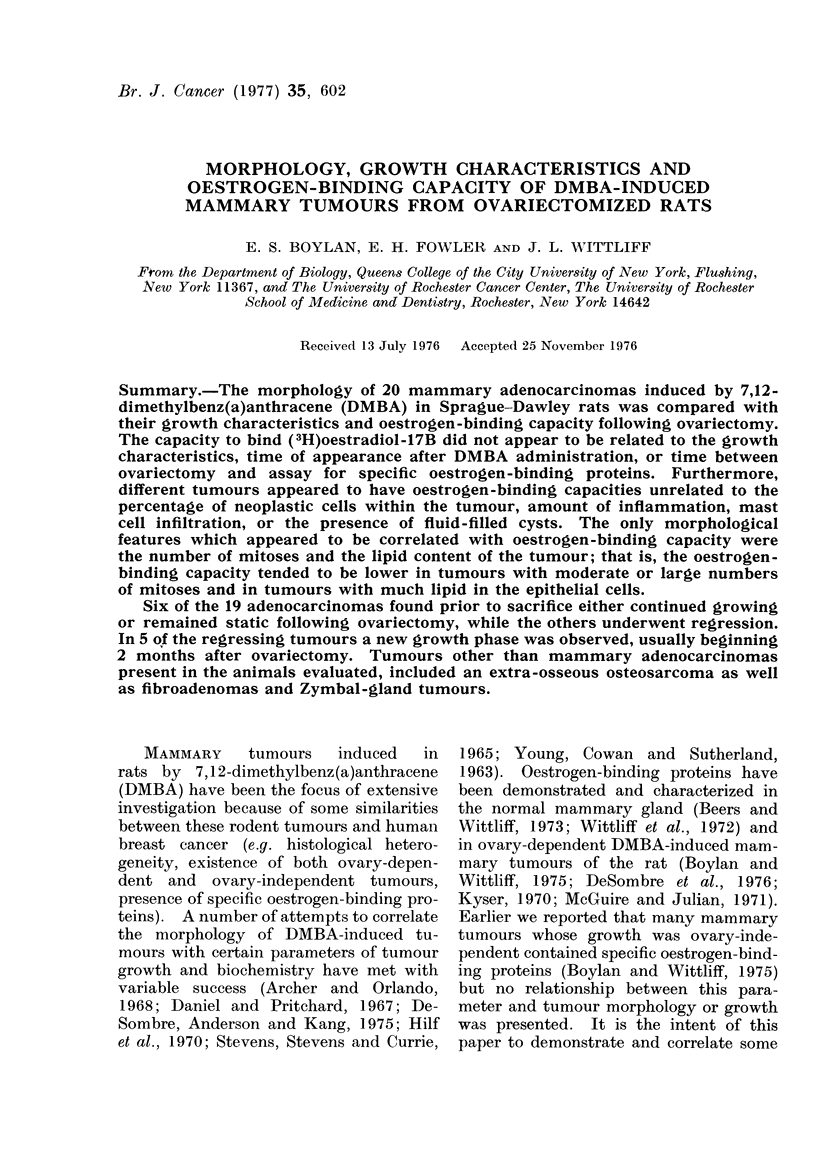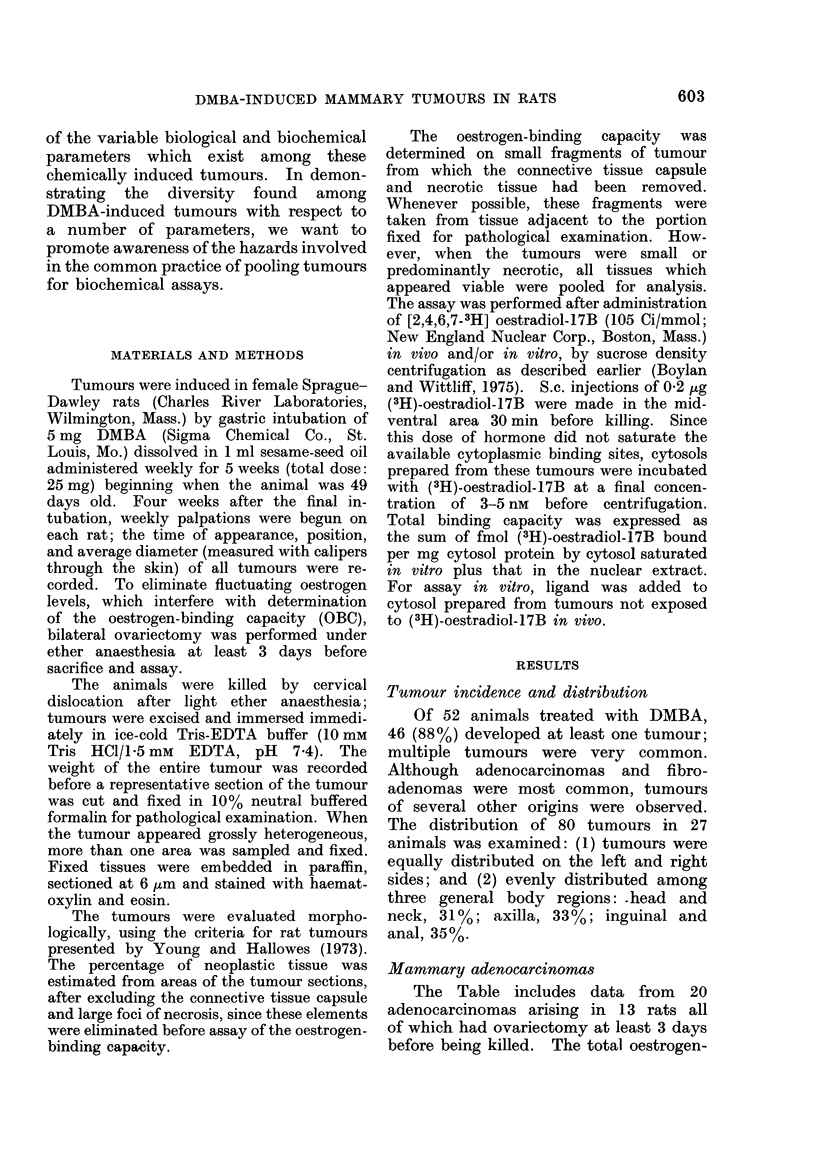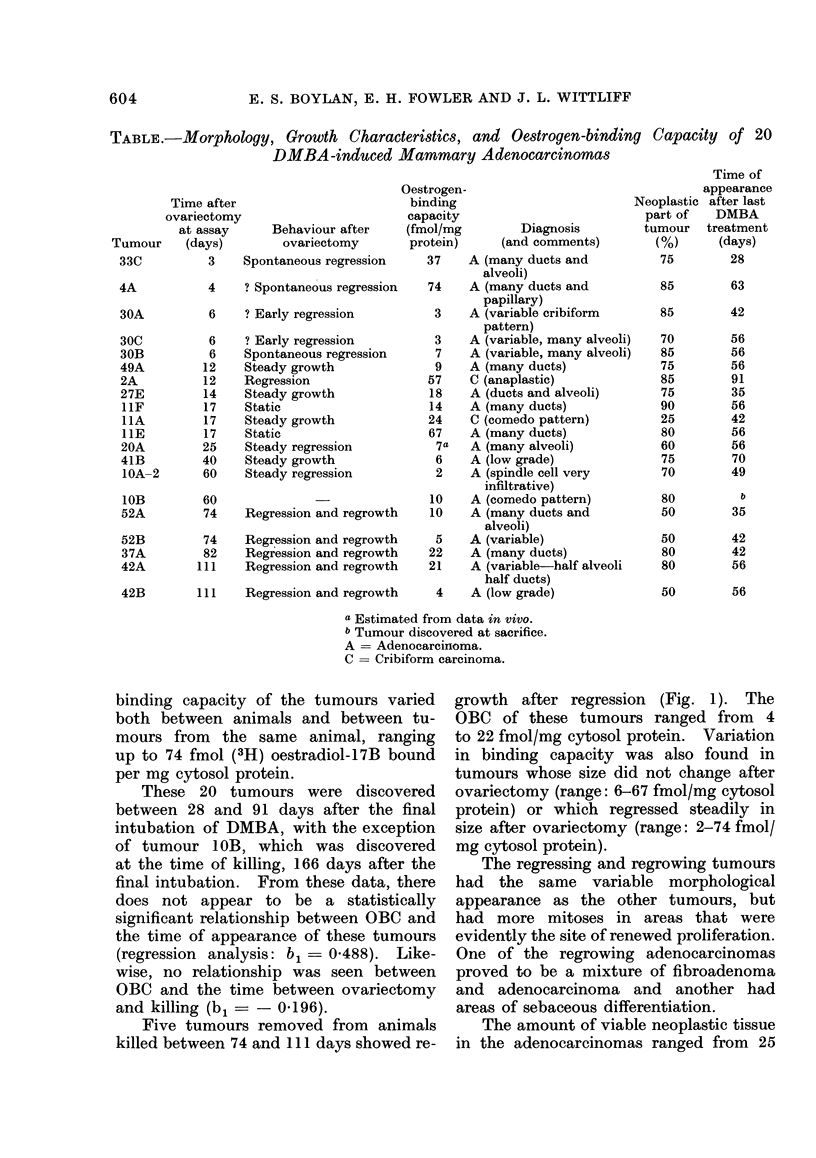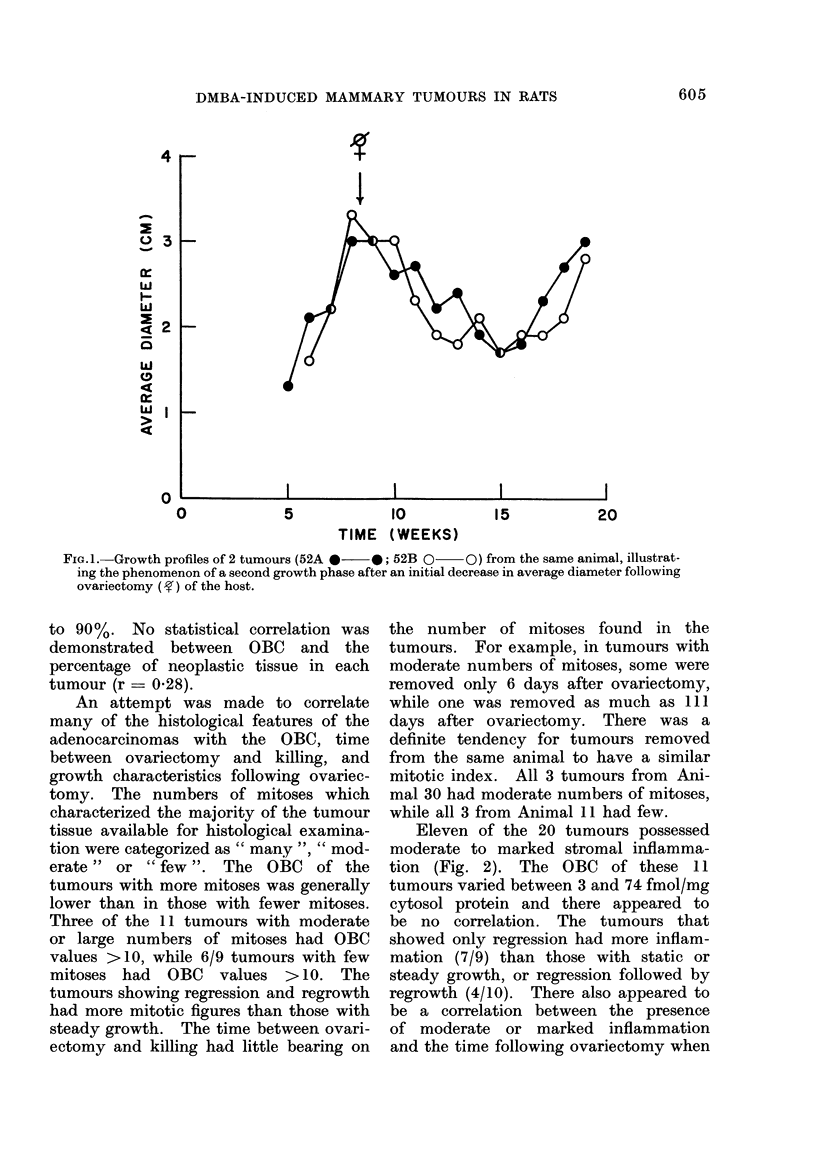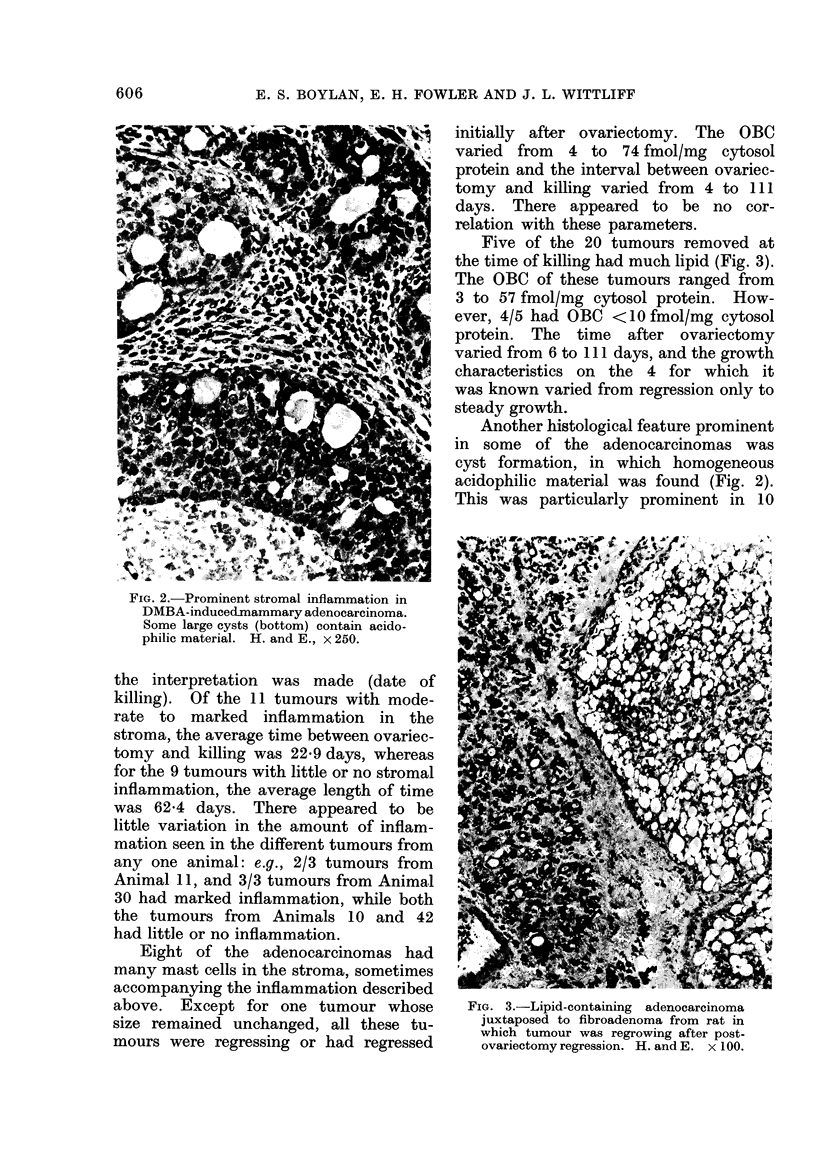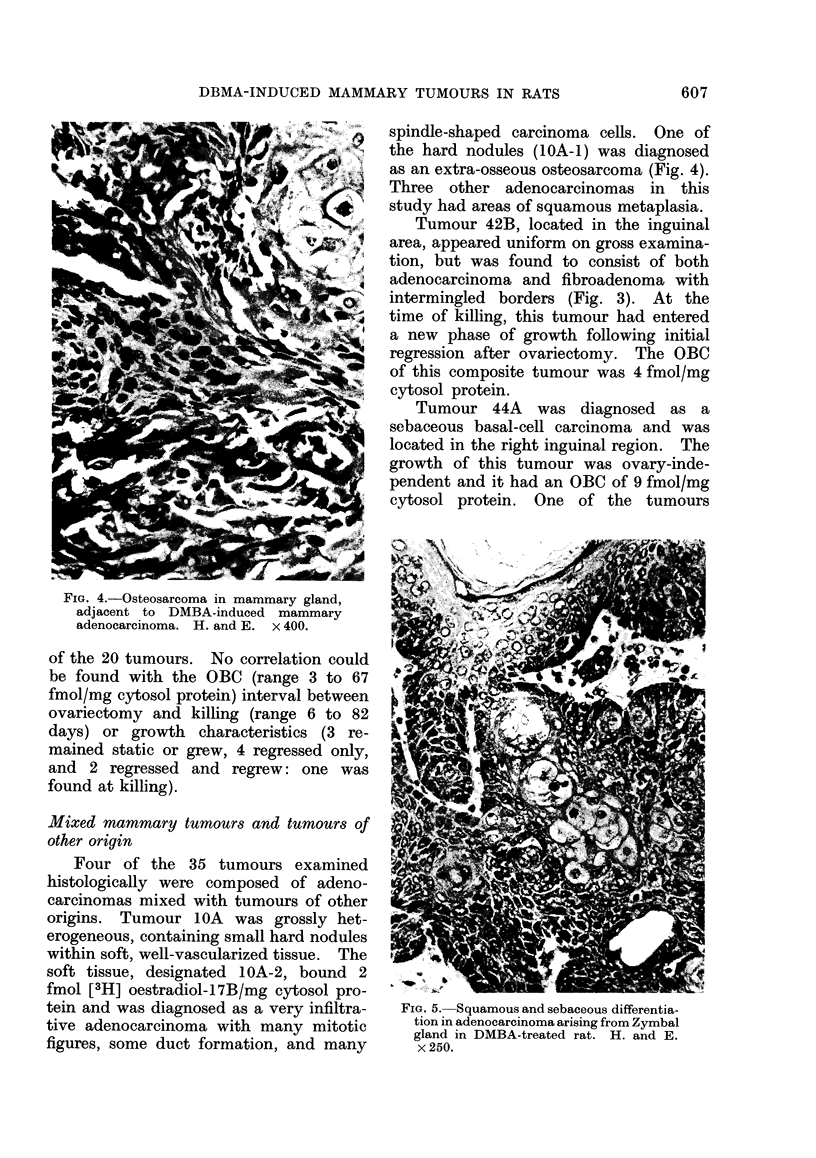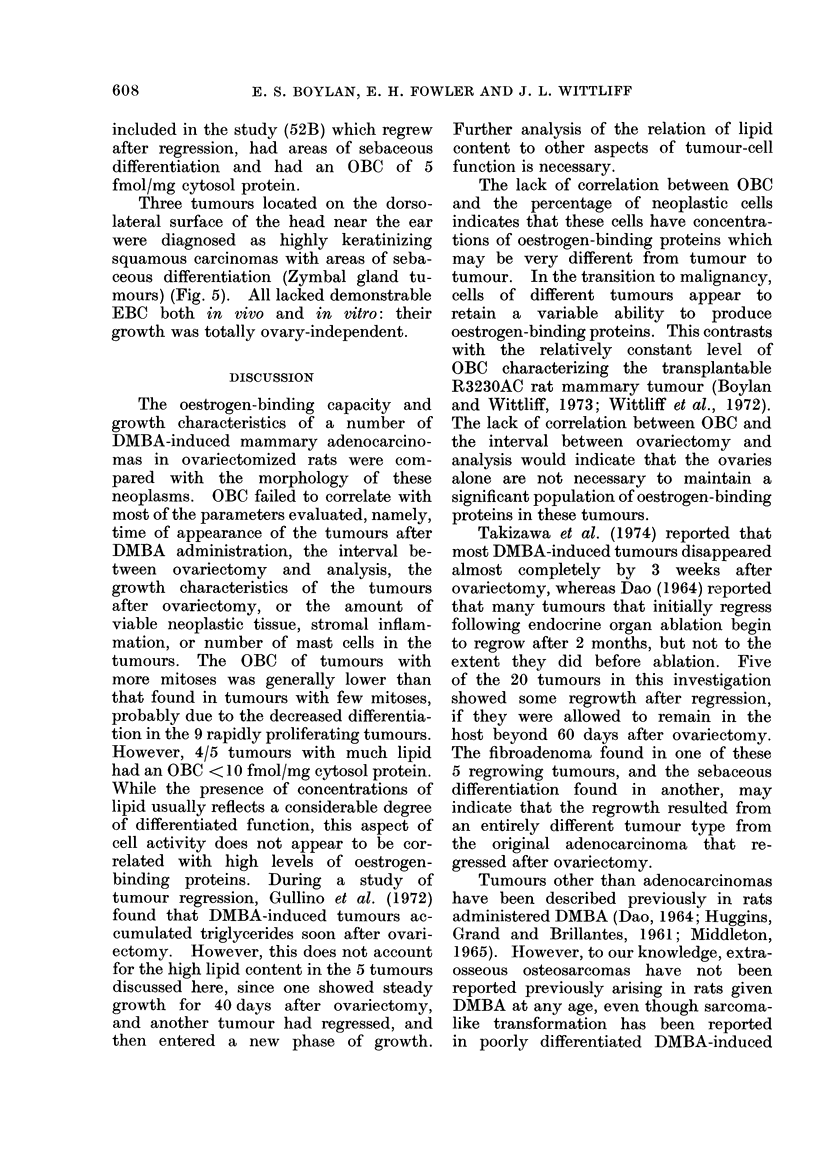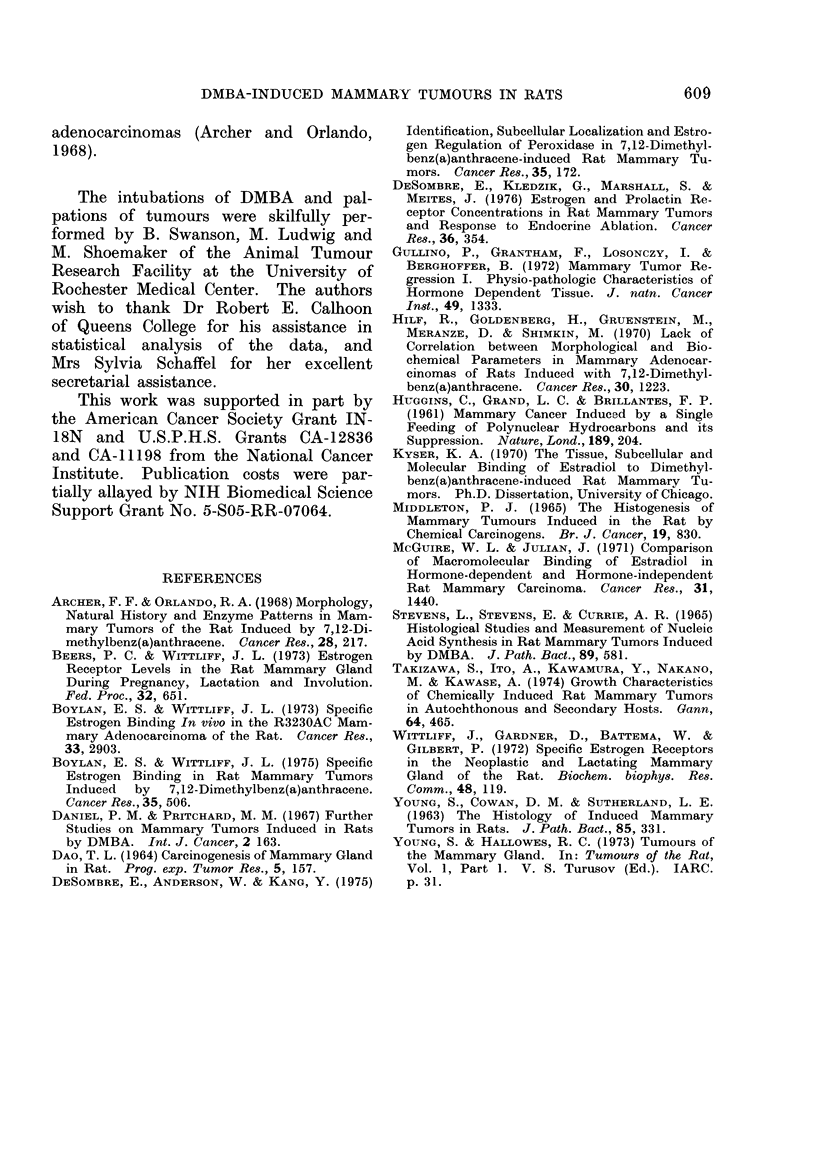# Morphology, growth characteristics and oestrogen-binding capacity of DMBA-induced mammary tumours from ovariectomized rats.

**DOI:** 10.1038/bjc.1977.94

**Published:** 1977-05

**Authors:** E. S. Boylan, E. H. Fowler, J. L. Wittliff

## Abstract

**Images:**


Br. J. Cancer (1977) 35, 602

### MORPHOLOGY, GROWTH CHARACTERISTICS AND OESTROGEN-BINDING CAPACITY OF DMBA-INDUCED MAMMARY TUMOURS FROM OVARIECTOMIZED RATS

E. S. BOYLAN, E. H. FOWLER AND J. L. WITTLIFF

*From the Department of Biology, Queens College of the City University of New York, Flushing, New York* 11367, *and The University of Rochester Cancer Center, The University of Rochester School of Medicine and Dentistry, Rochester, New York* 14642

Received 13 July 1976 Accepted 25 November 1976

**Summary.—The morphology of 20 mammary adenocarcinomas induced by 7,12-dimethylbenz(a)anthracene (DMBA) in Sprague–Dawley rats was compared with their growth characteristics and oestrogen-binding capacity following ovariectomy. The capacity to bind ($^3$H)oestradiol-17B did not appear to be related to the growth characteristics, time of appearance after DMBA administration, or time between ovariectomy and assay for specific oestrogen-binding proteins. Furthermore, different tumours appeared to have oestrogen-binding capacities unrelated to the percentage of neoplastic cells within the tumour, amount of inflammation, mast cell infiltration, or the presence of fluid-filled cysts. The only morphological features which appeared to be correlated with oestrogen-binding capacity were the number of mitoses and the lipid content of the tumour; that is, the oestrogen-binding capacity tended to be lower in tumours with moderate or large numbers of mitoses and in tumours with much lipid in the epithelial cells.**

**Six of the 19 adenocarcinomas found prior to sacrifice either continued growing or remained static following ovariectomy, while the others underwent regression. In 5 of the regressing tumours a new growth phase was observed, usually beginning 2 months after ovariectomy. Tumours other than mammary adenocarcinomas present in the animals evaluated, included an extra-osseous osteosarcoma as well as fibroadenomas and Zymbal-gland tumours.**

MAMMARY tumours induced in rats by 7,12-dimethylbenz(a)anthracene (DMBA) have been the focus of extensive investigation because of some similarities between these rodent tumours and human breast cancer (*e.g.* histological heterogeneity, existence of both ovary-dependent and ovary-independent tumours, presence of specific oestrogen-binding proteins). A number of attempts to correlate the morphology of DMBA-induced tumours with certain parameters of tumour growth and biochemistry have met with variable success (Archer and Orlando, 1968; Daniel and Pritchard, 1967; DeSombre, Anderson and Kang, 1975; Hilf *et al.*, 1970; Stevens, Stevens and Currie, 1965; Young, Cowan and Sutherland, 1963). Oestrogen-binding proteins have been demonstrated and characterized in the normal mammary gland (Beers and Wittliff, 1973; Wittliff *et al.*, 1972) and in ovary-dependent DMBA-induced mammary tumours of the rat (Boylan and Wittliff, 1975; DeSombre *et al.*, 1976; Kyser, 1970; McGuire and Julian, 1971). Earlier we reported that many mammary tumours whose growth was ovary-independent contained specific oestrogen-binding proteins (Boylan and Wittliff, 1975) but no relationship between this parameter and tumour morphology or growth was presented. It is the intent of this paper to demonstrate and correlate some of the variable biological and biochemical parameters which exist among these chemically induced tumours. In demonstrating the diversity found among DMBA-induced tumours with respect to a number of parameters, we want to promote awareness of the hazards involved in the common practice of pooling tumours for biochemical assays.

### MATERIALS AND METHODS

Tumours were induced in female Sprague–Dawley rats (Charles River Laboratories, Wilmington, Mass.) by gastric intubation of 5 mg DMBA (Sigma Chemical Co., St. Louis, Mo.) dissolved in 1 ml sesame-seed oil administered weekly for 5 weeks (total dose: 25 mg) beginning when the animal was 49 days old. Four weeks after the final intubation, weekly palpations were begun on each rat; the time of appearance, position, and average diameter (measured with calipers through the skin) of all tumours were recorded. To eliminate fluctuating oestrogen levels, which interfere with determination of the oestrogen-binding capacity (OBC), bilateral ovariectomy was performed under ether anaesthesia at least 3 days before sacrifice and assay.

The animals were killed by cervical dislocation after light ether anaesthesia; tumours were excised and immersed immediately in ice-cold Tris-EDTA buffer (10 mM Tris HCl/1·5 mM EDTA, pH 7·4). The weight of the entire tumour was recorded before a representative section of the tumour was cut and fixed in 10% neutral buffered formalin for pathological examination. When the tumour appeared grossly heterogeneous, more than one area was sampled and fixed. Fixed tissues were embedded in paraffin, sectioned at 6 $\mu$m and stained with haematoxylin and eosin.

The tumours were evaluated morphologically, using the criteria for rat tumours presented by Young and Hallowes (1973). The percentage of neoplastic tissue was estimated from areas of the tumour sections, after excluding the connective tissue capsule and large foci of necrosis, since these elements were eliminated before assay of the oestrogen-binding capacity.

The oestrogen-binding capacity was determined on small fragments of tumour from which the connective tissue capsule and necrotic tissue had been removed. Whenever possible, these fragments were taken from tissue adjacent to the portion fixed for pathological examination. However, when the tumours were small or predominantly necrotic, all tissues which appeared viable were pooled for analysis. The assay was performed after administration of [2,4,6,7-$^3$H] oestradiol-17B (105 Ci/mmol; New England Nuclear Corp., Boston, Mass.) *in vivo* and/or *in vitro*, by sucrose density centrifugation as described earlier (Boylan and Wittliff, 1975). S.c. injections of 0·2 $\mu$g ($^3$H)-oestradiol-17B were made in the mid-ventral area 30 min before killing. Since this dose of hormone did not saturate the available cytoplasmic binding sites, cytosols prepared from these tumours were incubated with ($^3$H)-oestradiol-17B at a final concentration of 3–5 nM before centrifugation. Total binding capacity was expressed as the sum of fmol ($^3$H)-oestradiol-17B bound per mg cytosol protein by cytosol saturated *in vitro* plus that in the nuclear extract. For assay *in vitro*, ligand was added to cytosol prepared from tumours not exposed to ($^3$H)-oestradiol-17B *in vivo*.

### RESULTS

### *Tumour incidence and distribution*

Of 52 animals treated with DMBA, 46 (88%) developed at least one tumour; multiple tumours were very common. Although adenocarcinomas and fibroadenomas were most common, tumours of several other origins were observed. The distribution of 80 tumours in 27 animals was examined: (1) tumours were equally distributed on the left and right sides; and (2) evenly distributed among three general body regions: head and neck, 31%; axilla, 33%; inguinal and anal, 35%.

### *Mammary adenocarcinomas*

The Table includes data from 20 adenocarcinomas arising in 13 rats all of which had ovariectomy at least 3 days before being killed. The total oestrogen-

TABLE.—*Morphology, Growth Characteristics, and Oestrogen-binding Capacity of* 20 *DMBA-induced Mammary Adenocarcinomas*

| Tumour | Time after ovariectomy at assay (days) | Behaviour after ovariectomy | Oestrogen-binding capacity (fmol/mg protein) | Diagnosis (and comments) | Neoplastic part of tumour (%) | Time of appearance after last DMBA treatment (days) |
|---|---|---|---|---|---|---|
| 33C | 3 | Spontaneous regression | 37 | A (many ducts and alveoli) | 75 | 28 |
| 4A | 4 | ? Spontaneous regression | 74 | A (many ducts and papillary) | 85 | 63 |
| 30A | 6 | ? Early regression | 3 | A (variable cribiform pattern) | 85 | 42 |
| 30C | 6 | ? Early regression | 3 | A (variable, many alveoli) | 70 | 56 |
| 30B | 6 | Spontaneous regression | 7 | A (variable, many alveoli) | 85 | 56 |
| 49A | 12 | Steady growth | 9 | A (many ducts) | 75 | 56 |
| 2A | 12 | Regression | 57 | C (anaplastic) | 85 | 91 |
| 27E | 14 | Steady growth | 18 | A (ducts and alveoli) | 75 | 35 |
| 11F | 17 | Static | 14 | A (many ducts) | 90 | 56 |
| 11A | 17 | Steady growth | 24 | C (comedo pattern) | 25 | 42 |
| 11E | 17 | Static | 67 | A (many ducts) | 80 | 56 |
| 20A | 25 | Steady regression | 7[a] | A (many alveoli) | 60 | 56 |
| 41B | 40 | Steady growth | 6 | A (low grade) | 75 | 70 |
| 10A-2 | 60 | Steady regression | 2 | A (spindle cell very infiltrative) | 70 | 49 |
| 10B | 60 | — | 10 | A (comedo pattern) | 80 | [b] |
| 52A | 74 | Regression and regrowth | 10 | A (many ducts and alveoli) | 50 | 35 |
| 52B | 74 | Regression and regrowth | 5 | A (variable) | 50 | 42 |
| 37A | 82 | Regression and regrowth | 22 | A (many ducts) | 80 | 42 |
| 42A | 111 | Regression and regrowth | 21 | A (variable—half alveoli half ducts) | 80 | 56 |
| 42B | 111 | Regression and regrowth | 4 | A (low grade) | 50 | 56 |

[a] Estimated from data *in vivo*.
[b] Tumour discovered at sacrifice.
A = Adenocarcinoma.
C = Cribiform carcinoma.

binding capacity of the tumours varied both between animals and between tumours from the same animal, ranging up to 74 fmol ($^3$H) oestradiol-17B bound per mg cytosol protein.

These 20 tumours were discovered between 28 and 91 days after the final intubation of DMBA, with the exception of tumour 10B, which was discovered at the time of killing, 166 days after the final intubation. From these data, there does not appear to be a statistically significant relationship between OBC and the time of appearance of these tumours (regression analysis: $b_1 = 0{\cdot}488$). Likewise, no relationship was seen between OBC and the time between ovariectomy and killing ($b_1 = -0{\cdot}196$).

Five tumours removed from animals killed between 74 and 111 days showed regrowth after regression (Fig. 1). The OBC of these tumours ranged from 4 to 22 fmol/mg cytosol protein. Variation in binding capacity was also found in tumours whose size did not change after ovariectomy (range: 6–67 fmol/mg cytosol protein) or which regressed steadily in size after ovariectomy (range: 2–74 fmol/mg cytosol protein).

The regressing and regrowing tumours had the same variable morphological appearance as the other tumours, but had more mitoses in areas that were evidently the site of renewed proliferation. One of the regrowing adenocarcinomas proved to be a mixture of fibroadenoma and adenocarcinoma and another had areas of sebaceous differentiation.

The amount of viable neoplastic tissue in the adenocarcinomas ranged from 25

FIG. 1.—Growth profiles of 2 tumours (52A ●——●; 52B ○——○) from the same animal, illustrating the phenomenon of a second growth phase after an initial decrease in average diameter following ovariectomy (⚲) of the host.

to 90%. No statistical correlation was demonstrated between OBC and the percentage of neoplastic tissue in each tumour (r = 0·28).

An attempt was made to correlate many of the histological features of the adenocarcinomas with the OBC, time between ovariectomy and killing, and growth characteristics following ovariectomy. The numbers of mitoses which characterized the majority of the tumour tissue available for histological examination were categorized as " many ", " moderate " or " few ". The OBC of the tumours with more mitoses was generally lower than in those with fewer mitoses. Three of the 11 tumours with moderate or large numbers of mitoses had OBC values >10, while 6/9 tumours with few mitoses had OBC values >10. The tumours showing regression and regrowth had more mitotic figures than those with steady growth. The time between ovariectomy and killing had little bearing on the number of mitoses found in the tumours. For example, in tumours with moderate numbers of mitoses, some were removed only 6 days after ovariectomy, while one was removed as much as 111 days after ovariectomy. There was a definite tendency for tumours removed from the same animal to have a similar mitotic index. All 3 tumours from Animal 30 had moderate numbers of mitoses, while all 3 from Animal 11 had few.

Eleven of the 20 tumours possessed moderate to marked stromal inflammation (Fig. 2). The OBC of these 11 tumours varied between 3 and 74 fmol/mg cytosol protein and there appeared to be no correlation. The tumours that showed only regression had more inflammation (7/9) than those with static or steady growth, or regression followed by regrowth (4/10). There also appeared to be a correlation between the presence of moderate or marked inflammation and the time following ovariectomy when

FIG. 2.—Prominent stromal inflammation in DMBA-inducedmammary adenocarcinoma. Some large cysts (bottom) contain acidophilic material. H. and E., ×250.

the interpretation was made (date of killing). Of the 11 tumours with moderate to marked inflammation in the stroma, the average time between ovariectomy and killing was 22·9 days, whereas for the 9 tumours with little or no stromal inflammation, the average length of time was 62·4 days. There appeared to be little variation in the amount of inflammation seen in the different tumours from any one animal: *e.g.*, 2/3 tumours from Animal 11, and 3/3 tumours from Animal 30 had marked inflammation, while both the tumours from Animals 10 and 42 had little or no inflammation.

Eight of the adenocarcinomas had many mast cells in the stroma, sometimes accompanying the inflammation described above. Except for one tumour whose size remained unchanged, all these tumours were regressing or had regressed initially after ovariectomy. The OBC varied from 4 to 74 fmol/mg cytosol protein and the interval between ovariectomy and killing varied from 4 to 111 days. There appeared to be no correlation with these parameters.

Five of the 20 tumours removed at the time of killing had much lipid (Fig. 3). The OBC of these tumours ranged from 3 to 57 fmol/mg cytosol protein. However, 4/5 had OBC <10 fmol/mg cytosol protein. The time after ovariectomy varied from 6 to 111 days, and the growth characteristics on the 4 for which it was known varied from regression only to steady growth.

Another histological feature prominent in some of the adenocarcinomas was cyst formation, in which homogeneous acidophilic material was found (Fig. 2). This was particularly prominent in 10

FIG. 3.—Lipid-containing adenocarcinoma juxtaposed to fibroadenoma from rat in which tumour was regrowing after post-ovariectomy regression. H. and E. ×100.

FIG. 4.—Osteosarcoma in mammary gland, adjacent to DMBA-induced mammary adenocarcinoma. H. and E. ×400.

of the 20 tumours. No correlation could be found with the OBC (range 3 to 67 fmol/mg cytosol protein) interval between ovariectomy and killing (range 6 to 82 days) or growth characteristics (3 remained static or grew, 4 regressed only, and 2 regressed and regrew: one was found at killing).

*Mixed mammary tumours and tumours of other origin*

Four of the 35 tumours examined histologically were composed of adenocarcinomas mixed with tumours of other origins. Tumour 10A was grossly heterogeneous, containing small hard nodules within soft, well-vascularized tissue. The soft tissue, designated 10A-2, bound 2 fmol [$^3$H] oestradiol-17B/mg cytosol protein and was diagnosed as a very infiltrative adenocarcinoma with many mitotic figures, some duct formation, and many spindle-shaped carcinoma cells. One of the hard nodules (10A-1) was diagnosed as an extra-osseous osteosarcoma (Fig. 4). Three other adenocarcinomas in this study had areas of squamous metaplasia.

Tumour 42B, located in the inguinal area, appeared uniform on gross examination, but was found to consist of both adenocarcinoma and fibroadenoma with intermingled borders (Fig. 3). At the time of killing, this tumour had entered a new phase of growth following initial regression after ovariectomy. The OBC of this composite tumour was 4 fmol/mg cytosol protein.

Tumour 44A was diagnosed as a sebaceous basal-cell carcinoma and was located in the right inguinal region. The growth of this tumour was ovary-independent and it had an OBC of 9 fmol/mg cytosol protein. One of the tumours

FIG. 5.—Squamous and sebaceous differentiation in adenocarcinoma arising from Zymbal gland in DMBA-treated rat. H. and E. ×250.

included in the study (52B) which regrew after regression, had areas of sebaceous differentiation and had an OBC of 5 fmol/mg cytosol protein.

Three tumours located on the dorsolateral surface of the head near the ear were diagnosed as highly keratinizing squamous carcinomas with areas of sebaceous differentiation (Zymbal gland tumours) (Fig. 5). All lacked demonstrable EBC both *in vivo* and *in vitro*: their growth was totally ovary-independent.

### DISCUSSION

The oestrogen-binding capacity and growth characteristics of a number of DMBA-induced mammary adenocarcinomas in ovariectomized rats were compared with the morphology of these neoplasms. OBC failed to correlate with most of the parameters evaluated, namely, time of appearance of the tumours after DMBA administration, the interval between ovariectomy and analysis, the growth characteristics of the tumours after ovariectomy, or the amount of viable neoplastic tissue, stromal inflammation, or number of mast cells in the tumours. The OBC of tumours with more mitoses was generally lower than that found in tumours with few mitoses, probably due to the decreased differentiation in the 9 rapidly proliferating tumours. However, 4/5 tumours with much lipid had an OBC $<10$ fmol/mg cytosol protein. While the presence of concentrations of lipid usually reflects a considerable degree of differentiated function, this aspect of cell activity does not appear to be correlated with high levels of oestrogen-binding proteins. During a study of tumour regression, Gullino *et al.* (1972) found that DMBA-induced tumours accumulated triglycerides soon after ovariectomy. However, this does not account for the high lipid content in the 5 tumours discussed here, since one showed steady growth for 40 days after ovariectomy, and another tumour had regressed, and then entered a new phase of growth. Further analysis of the relation of lipid content to other aspects of tumour-cell function is necessary.

The lack of correlation between OBC and the percentage of neoplastic cells indicates that these cells have concentrations of oestrogen-binding proteins which may be very different from tumour to tumour. In the transition to malignancy, cells of different tumours appear to retain a variable ability to produce oestrogen-binding proteins. This contrasts with the relatively constant level of OBC characterizing the transplantable R3230AC rat mammary tumour (Boylan and Wittliff, 1973; Wittliff *et al.*, 1972). The lack of correlation between OBC and the interval between ovariectomy and analysis would indicate that the ovaries alone are not necessary to maintain a significant population of oestrogen-binding proteins in these tumours.

Takizawa *et al.* (1974) reported that most DMBA-induced tumours disappeared almost completely by 3 weeks after ovariectomy, whereas Dao (1964) reported that many tumours that initially regress following endocrine organ ablation begin to regrow after 2 months, but not to the extent they did before ablation. Five of the 20 tumours in this investigation showed some regrowth after regression, if they were allowed to remain in the host beyond 60 days after ovariectomy. The fibroadenoma found in one of these 5 regrowing tumours, and the sebaceous differentiation found in another, may indicate that the regrowth resulted from an entirely different tumour type from the original adenocarcinoma that regressed after ovariectomy.

Tumours other than adenocarcinomas have been described previously in rats administered DMBA (Dao, 1964; Huggins, Grand and Brillantes, 1961; Middleton, 1965). However, to our knowledge, extra-osseous osteosarcomas have not been reported previously arising in rats given DMBA at any age, even though sarcoma-like transformation has been reported in poorly differentiated DMBA-induced adenocarcinomas (Archer and Orlando, 1968).

The intubations of DMBA and palpations of tumours were skilfully performed by B. Swanson, M. Ludwig and M. Shoemaker of the Animal Tumour Research Facility at the University of Rochester Medical Center. The authors wish to thank Dr Robert E. Calhoon of Queens College for his assistance in statistical analysis of the data, and Mrs Sylvia Schaffel for her excellent secretarial assistance.

This work was supported in part by the American Cancer Society Grant IN-18N and U.S.P.H.S. Grants CA-12836 and CA-11198 from the National Cancer Institute. Publication costs were partially allayed by NIH Biomedical Science Support Grant No. 5-S05-RR-07064.

### REFERENCES

Archer, F. F. & Orlando, R. A. (1968) Morphology, Natural History and Enzyme Patterns in Mammary Tumors of the Rat Induced by 7,12-Dimethylbenz(a)anthracene. *Cancer Res.*, **28**, 217.

Beers, P. C. & Wittliff, J. L. (1973) Estrogen Receptor Levels in the Rat Mammary Gland During Pregnancy, Lactation and Involution. *Fed. Proc.*, **32**, 651.

Boylan, E. S. & Wittliff, J. L. (1973) Specific Estrogen Binding *In vivo* in the R3230AC Mammary Adenocarcinoma of the Rat. *Cancer Res.*, **33**, 2903.

Boylan, E. S. & Wittliff, J. L. (1975) Specific Estrogen Binding in Rat Mammary Tumors Induced by 7,12-Dimethylbenz(a)anthracene. *Cancer Res.*, **35**, 506.

Daniel, P. M. & Pritchard, M. M. (1967) Further Studies on Mammary Tumors Induced in Rats by DMBA. *Int. J. Cancer*, **2** 163.

Dao, T. L. (1964) Carcinogenesis of Mammary Gland in Rat. *Prog. exp. Tumor Res.*, **5**, 157.

DeSombre, E., Anderson, W. & Kang, Y. (1975) Identification, Subcellular Localization and Estrogen Regulation of Peroxidase in 7,12-Dimethylbenz(a)anthracene-induced Rat Mammary Tumors. *Cancer Res.*, **35**, 172.

DeSombre, E., Kledzik, G., Marshall, S. & Meites, J. (1976) Estrogen and Prolactin Receptor Concentrations in Rat Mammary Tumors and Response to Endocrine Ablation. *Cancer Res.*, **36**, 354.

Gullino, P., Grantham, F., Losonczy, I. & Berghoffer, B. (1972) Mammary Tumor Regression I. Physio-pathologic Characteristics of Hormone Dependent Tissue. *J. natn. Cancer Inst.*, **49**, 1333.

Hilf, R., Goldenberg, H., Gruenstein, M., Meranze, D. & Shimkin, M. (1970) Lack of Correlation between Morphological and Biochemical Parameters in Mammary Adenocarcinomas of Rats Induced with 7,12-Dimethylbenz(a)anthracene. *Cancer Res.*, **30**, 1223.

Huggins, C., Grand, L. C. & Brillantes, F. P. (1961) Mammary Cancer Induced by a Single Feeding of Polynuclear Hydrocarbons and its Suppression. *Nature, Lond.*, **189**, 204.

Kyser, K. A. (1970) The Tissue, Subcellular and Molecular Binding of Estradiol to Dimethylbenz(a)anthracene-induced Rat Mammary Tumors. Ph.D. Dissertation, University of Chicago.

Middleton, P. J. (1965) The Histogenesis of Mammary Tumours Induced in the Rat by Chemical Carcinogens. *Br. J. Cancer*, **19**, 830.

McGuire, W. L. & Julian, J. (1971) Comparison of Macromolecular Binding of Estradiol in Hormone-dependent and Hormone-independent Rat Mammary Carcinoma. *Cancer Res.*, **31**, 1440.

Stevens, L., Stevens, E. & Currie, A. R. (1965) Histological Studies and Measurement of Nucleic Acid Synthesis in Rat Mammary Tumors Induced by DMBA. *J. Path. Bact.*, **89**, 581.

Takizawa, S., Ito, A., Kawamura, Y., Nakano, M. & Kawase, A. (1974) Growth Characteristics of Chemically Induced Rat Mammary Tumors in Autochthonous and Secondary Hosts. *Gann*, **64**, 465.

Wittliff, J., Gardner, D., Battema, W. & Gilbert, P. (1972) Specific Estrogen Receptors in the Neoplastic and Lactating Mammary Gland of the Rat. *Biochem. biophys. Res. Comm.*, **48**, 119.

Young, S., Cowan, D. M. & Sutherland, L. E. (1963) The Histology of Induced Mammary Tumors in Rats. *J. Path. Bact.*, **85**, 331.

Young, S. & Hallowes, R. C. (1973) Tumours of the Mammary Gland. In: *Tumours of the Rat*, Vol. 1, Part 1. V. S. Turusov (Ed.). IARC. p. 31.